# MOVING: A Multi-Modal Dataset of EEG Signals and Virtual Glove Hand Tracking

**DOI:** 10.3390/s24165207

**Published:** 2024-08-11

**Authors:** Enrico Mattei, Daniele Lozzi, Alessandro Di Matteo, Alessia Cipriani, Costanzo Manes, Giuseppe Placidi

**Affiliations:** 1 A^2^VI-Lab, Department of Life, Health and Environmental Sciences, University of L’Aquila, 67100 L’Aquila, Italy; daniele.lozzi@graduate.univaq.it (D.L.); alessandro.dimatteo1@graduate.univaq.it (A.D.M.); alessia.cipriani03@icatt.it (A.C.); giuseppe.placidi@univaq.it (G.P.); 2 Department of Information Engineering, Computer Science and Mathematics, University of L’Aquila, 67100 L’Aquila, Italy; costanzo.manes@univaq.it; 3 Department of Diagnostic Imaging, Oncologic Radiotherapy and Hematology, Università Cattolica del Sacro Cuore, 00168 Rome, Italy

**Keywords:** BCI, EEG, virtual glove, EEGnetV4, dataset

## Abstract

Brain–computer interfaces (BCIs) are pivotal in translating neural activities into control commands for external assistive devices. Non-invasive techniques like electroencephalography (EEG) offer a balance of sensitivity and spatial-temporal resolution for capturing brain signals associated with motor activities. This work introduces MOVING, a Multi-Modal dataset of EEG signals and Virtual Glove Hand Tracking. This dataset comprises neural EEG signals and kinematic data associated with three hand movements—open/close, finger tapping, and wrist rotation—along with a rest period. The dataset, obtained from 11 subjects using a 32-channel dry wireless EEG system, also includes synchronized kinematic data captured by a Virtual Glove (VG) system equipped with two orthogonal Leap Motion Controllers. The use of these two devices allows for fast assembly (∼1 min), although introducing more noise than the gold standard devices for data acquisition. The study investigates which frequency bands in EEG signals are the most informative for motor task classification and the impact of baseline reduction on gesture recognition. Deep learning techniques, particularly EEGnetV4, are applied to analyze and classify movements based on the EEG data. This dataset aims to facilitate advances in BCI research and in the development of assistive devices for people with impaired hand mobility. This study contributes to the repository of EEG datasets, which is continuously increasing with data from other subjects, which is hoped to serve as benchmarks for new BCI approaches and applications.

## 1. Introduction

Research on brain waves for human–machine interaction began with the initial publication analyzing EEG data related to movement in 1968 [1]. The field of brain–computer interface (BCI) [2] studies focuses on recognizing mental conditions, such as emotions [3], mental workloads [4], and movements [5,6] (upper limb, wrist, and fingers) from human neurophysiological signals to control external assistive devices [7]. Brain signals acquired through acquisition processes are classified as invasive or non-invasive [8]. The non-invasive class includes electroencephalography (EEG), functional magnetic resonance imaging (fMRI), magnetoencephalography (MEG), and near-infrared spectroscopy (NIRS). Among these, EEG strikes a good mix between sensitivity and spatial-temporal resolution for measuring and analyzing movements. In the literature, EEG-based BCI is used to decode a user’s movement intention based on signs of active brain engagement in the preparation of desired movement paradigms known as motor imagery (MI), as well as to decode real human movements, i.e., Motor Execution (ME), using Artificial Intelligence (AI) approaches. To classify MI movement, deep learning (DL) algorithms must be trained using MI EEG datasets and then used as benchmarks before single subject data testing or to directly evaluate the suggested approaches. Many MI datasets are accessible in the literature. The EEG BCI dataset [9] was constructed by capturing signals from electrodes F3, F4, FC5, and FC6 from the Emotiv Device, and selecting four individuals to imagine the opening/closing of the right or left hand after the video stimuli for a total of 2400 trials. The EEG Motor Movement/Imagery Dataset [10] was developed by obtaining signals from the BCI2000 64-channel system by choosing 109 volunteer individuals who imagined opening and closing left or right hand, or opening and closing both hands or both feet according to video stimuli. The BCI Competition III dataset IIIa [11] has four classes (left hand, right hand, foot, tongue) acquired from three subjects by obtaining signals from a 64-channel Neuroscan EEG amplifier for a total of 60 trials per class. The BCI Competition III dataset IVa [12] was recorded with multichannel EEG amplifiers using 64 or 128 channels from ten healthy subjects. Subjects sat in a comfortable chair with arms resting on armrests and imagined left or right hand or foot or imagination of a visual (with eyes open), auditory, or tactile sensation. Moreover, BCI Competition IV dataset 2a [13] and 2b [14] are also available for MI. For the classification, many DL techniques are used. A deep metric model with a triple convolutional neural network (CNN) was used to classify MI of the right or left hand based on incoming visual stimuli; in the absence of a stimulus, it is deemed rest [15]. Electroencephalography topographical representation (ETR)-CNN classifies five MI movements and one resting movement using the ETR manipulation on the signals of the BCI Competition IV-2a dataset [16]. Classification Network-based long short-term memory (LSTM) is applied on the BCI Competition IV-2a dataset and to make the classification more resilient on the dataset’s signals, a one-dimensional-aggregate approximation is performed [17], as well as in other BCI domains such as emotions [18,19]. In a recent review [20], many EEG motor-related datasets are summarized and many of them are related to MI tasks, while few are related to ME. The distinction between MI and ME tasks is a well-known concept in scientific literature, and can be identified using neuroimaging methods in several experimental works [21,22] and in a comprehensive review [23]. Regarding ME, the approach used is the same as above for MI. Datasets, available in the literature, collect many types of real movements ranging from the most complex to the most intuitive, and DL techniques are applied to classify them. EEG Data for Voluntary Finger Tapping Movement is a collection of EEG data acquired during voluntary asynchronous index finger tapping by 14 healthy adults. EEG was recorded using a TruScan Deymed amplifier with 19 channels for three conditions: right finger tap, left finger tap, and resting state, with a sampling rate of 1024 Hz. Each participant performed 120 trials, 40 for each of the three conditions [24]. The EEG Motor Movement/Imagery Dataset has a section where the imagined movements, explained before, are replicated with the real movement of the subject [10]. The High-Gamma Dataset is a 128-electrode dataset collected from 14 healthy subjects during four-second trials of executed movements, separated into 13 runs per subject. Movements can be classified into four types: left hand, right hand, both feet, or rest [25]. The Upper Limb movements dataset [20] consists of data from 15 healthy subjects, of whom all except one were right-handed. To avoid muscle fatigue, subjects sat in a chair with their right arm fully supported by an exoskeleton. Each subject had two sessions on different days, no more than one week apart. Subjects conducted ME in the first session and MI in the second. Six different movements were executed with the right upper limb, beginning in a neutral posture. Additionally, a rest class was recorded in which individuals were advised to avoid moving and keep their starting position.

The present work is focused on creating a multi-modal EEG dataset to classify the neural signals and to correlate neural information with three human-hand movements (open/close, finger tapping, and wrist rotation) modeled by a CV-based system named Virtual Glove (VG) [26,27,28]. The conceptualization of this dataset follows the multi-modal approach of some previous works [29], but differentiated by using VG with two sensors for kinematic data acquisition at high precision. Furthermore, this article focuses on baseline analysis and the most informative frequency bands for motion classification using a deep learning technique operating on the power spectral density (PSD) [30]. The use of these two devices, VG and Enobio® EEG dry, allow data acquisition to begin in ∼1 min, thus making the device effectively usable in an outdoor environment. However, dry electrodes introduce significantly higher noise than wet EEG devices [31]; the latter are considered the gold standard for EEG recording [32]. The acquisition modality, the acquired data, and the classification movement process using the DL architecture EEGnetv4 [33] are detailed. This architecture has been developed to be useful in many BCI applications such as P300 cortical potentials movement-related and sensory-motor rhythms [34]. The protocol is carried out using a simple motor task, composed of sequences of hand movements tasks composed of MI action or ME. In the proposed work, according to the literature, three different movements (both MI and ME) are used: open/close, finger tapping, and wrist rotation. In addition, EEG data are recorded under rest conditions. The same movements performed in MI were followed by ME and rest for 10 min. The EEGnetV4, developed in the Pytorch environment, is trained and validated using this new dataset based on EEG signals in ME and rest conditions. The article is structured as follows: Section 2 details how the data were collected and modified, as well as the EEGnetV4 model used to classify the movements; Section 3 reports on the experimental work and discusses the results, Section 4 reports the discussion, and Section 5 is the conclusion.

## 2. Methodology

This work aims to present a multi-modal dataset on MI/ME on kinematic and brain data acquired with VG and a wireless dry, fast-wearable EEG device (Enobio^®^ EEG (https://www.neuroelectrics.com/solutions/enobio/32, accessed on 15 June 2024)). In addition, its goal is to analyze which frequency bands are more informative for ME classification and the role of the baseline reduction method. These devices were used in a semi-controlled environment to emulate real-life situations [35] where high noise and disturbances are present. The classification is performed with EEGnetV4 [33] because recent studies have shown that this architecture is one of the best performing [30,36]. Three classes of data were collected in both the ME and MI conditions: open/close, finger tapping, wrist rotation, and the rest condition using a dry EEG device and VG. These findings are made available as a starting point for the development of rehabilitation support systems for patients who have experienced hand injuries with loss of mobility. The EEGnetV4 model acts on the collected EEG signals to recognize movements, even if not executed correctly, allowing the corresponding movements to reproduce on a robot arm. In this way, it allows the patient to have feedback for improving the motor skills.

### 2.1. Data Structure

The dataset is composed of two main independent data structures: the EEG data and the VG data.

#### 2.1.1. EEG Data

**Figure 1:**
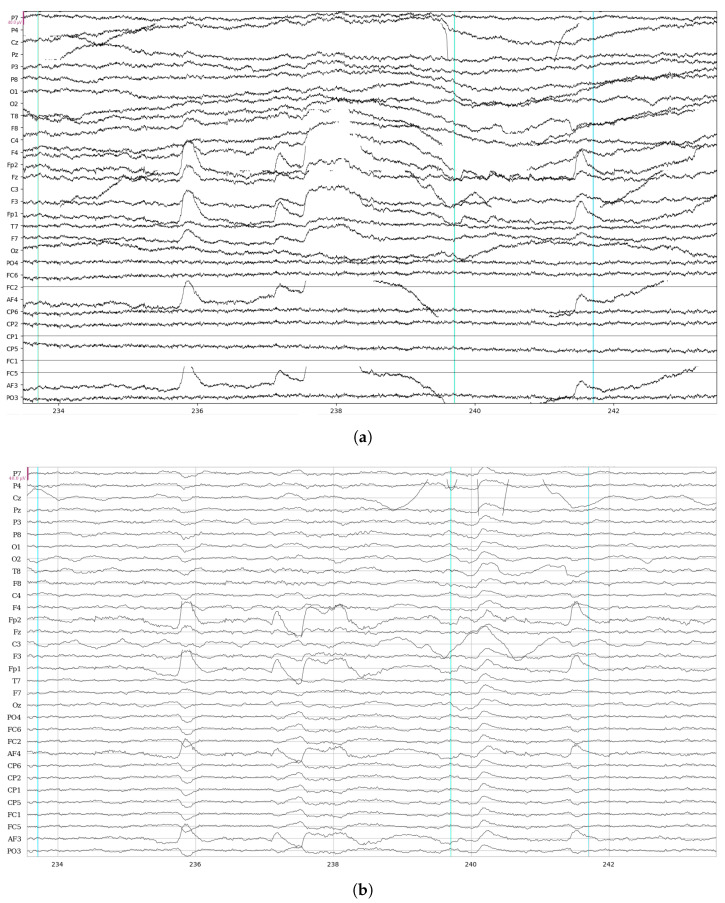
EEG raw data before (**a**) and after (**b**) a cleaning band-pass [1–45 Hz] filtering. In (**a**), the high noise level makes it difficult to visualize high-amplitude brain signals. Vertical lines represent the triggers for rest, fixation cross, and open/close movement, respectively.

The EEG signal is arranged as a 2D matrix, with rows representing channels (electrodes) and columns indicating time points (samples). The EEG recording involves 32 channels at 500 Hz throughout ∼10 min, thus generating a matrix ∼32 × 300,000. For the epoch creation, it is necessary to fix some markers to identify the occurring events. The markers are placed through synchronization between Unity (https://unity.com/, accessed on 15 June 2024), which communicates with the VG, and the EEG software, NIC2, that saves the EEG signal. In general, events are a matrix in which each row represents an event associated with the timestamp and the label of the event. This configuration, along with metadata such as sampling rate, electrode positions, and event markers, enables in-depth analysis and understanding of brain activity. An example of the data collected with this structure is shown in Figure 1.

#### 2.1.2. Virtual Glove Data

**Figure 2:**
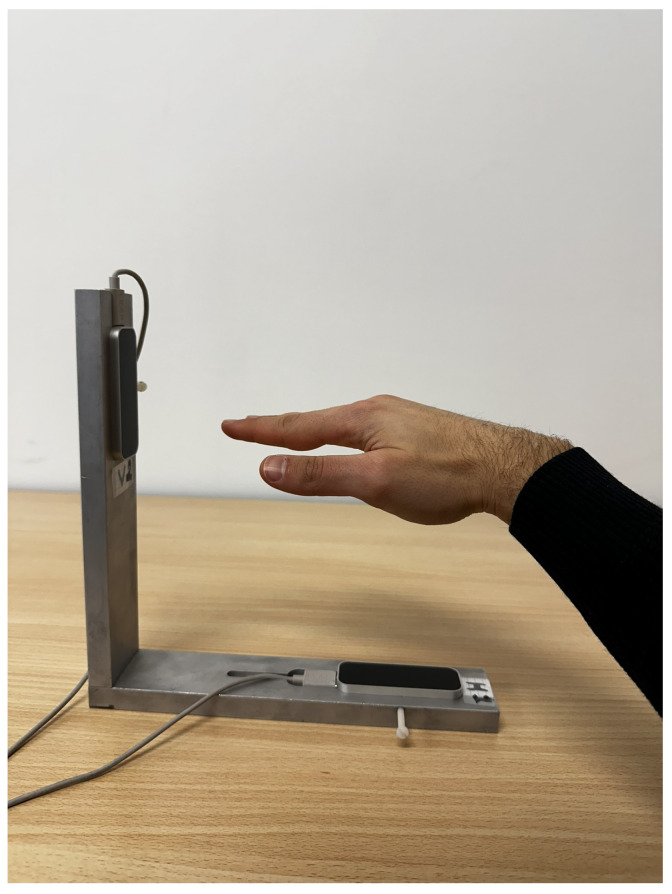
VG system while tracking the hand movements. The hand positioning system is united with the one of the VG.

**Figure 3:**
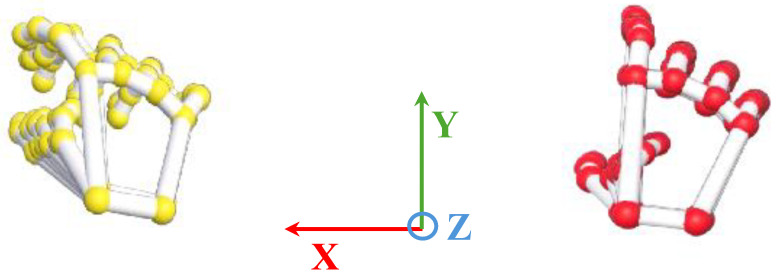
Model based on data collected by VG. The left part shows the vertical view; the right part shows the horizontal view. The Cartesian reference system is reported in the center.

VG uses two Leap Motion Controllers (LMCs), each generating a dynamic 4D numerical representation of the hand. A picture of the VG is shown in Figure 2. The hand model is reproduced into frames, each frame representing a temporal snapshot of the hand at a certain point in time (Figure 3). These frames collect detailed information on the recognized hands, including the precise positioning of 25 joints and their timestamp. Each joint represents the articulation between two bones or a terminal bone (fingertip) and provides precise coordinates in three dimensions (x, y, and z), as well as the calculation of velocity and acceleration. In addition to joint data, the system collects other hand-related information, such as the position and velocity of the palm. Furthermore, the VG was calibrated to a single reference system [37], and Figure 3 shows the basis vector of the VG. The former points from the palm to the fingers, whereas the latter indicates the palm’s direction related to the controller. These vectors are used to calculate the inner product and create a rotation matrix for the hand. This matrix simplifies the calculation of the hand’s orientation angles (yaw, pitch, and roll), as well as the angle between any two bones. Velocity and acceleration are also determined for each joint by combining neighboring frames. Finally, the two LMCs have been calibrated to guarantee that the views from both LMCs are aligned, hence improving data integration, and the data provided by the optimal viewer is used based on roll angle. This algorithm ensures that the data from the optimal viewer are used all the time [37].

### 2.2. Data Acquisition

**Figure 4:**
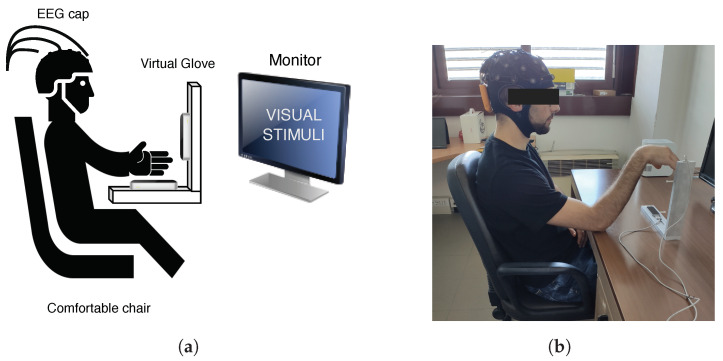
Acquisition environment scheme (**a**) and real acquisition environment (**b**) used in this work.

The protocol used in this work is similar to [24], in which the participant was seated in a comfortable chair, in front of a 20″ screen following the instructions visualized on the monitor. The participant wears an Enobio® EEG system with 32 dry electrodes (P7, P4, Cz, Pz, P3, P8, O1, O2, T8, F8, C4, F4, Fp2, Fz, C3, F3, Fp1, T7, F7, Oz, PO4, FC6, FC2, AF4, CP6, CP2, CP1, CP5, FC1, FC5, AF3, PO3), while the VG device is placed under the right arm to record kinematic data. Figure 4a depicts the experimental procedure and Figure 4b shows the acquisition environment.

**Figure 5:**
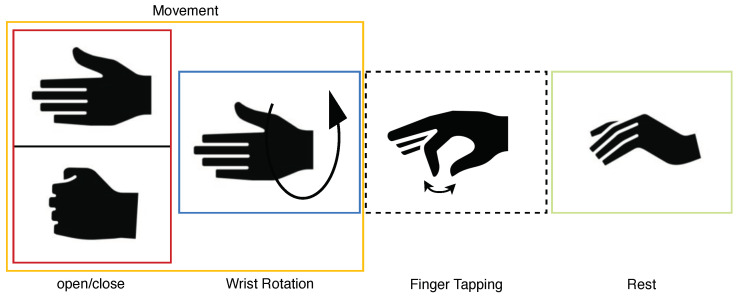
The analyzed movements. The dotted line represents the movement acquired but not analyzed in this work. The class “movement” is created by merging the open/close and wrist rotation classes.

**Figure 6:**
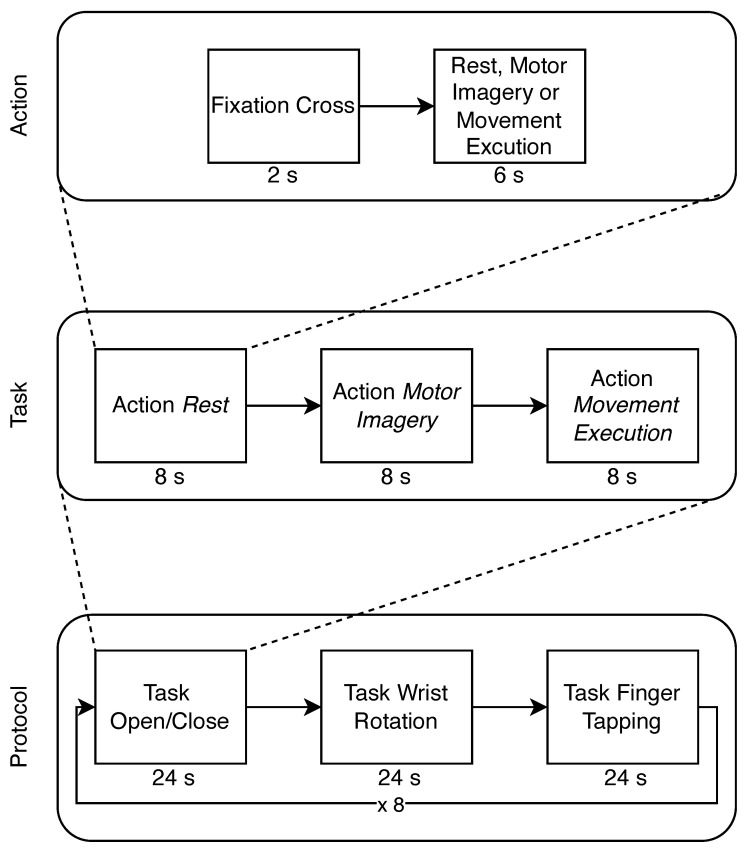
The used hand movement protocol.

**Figure 7:**
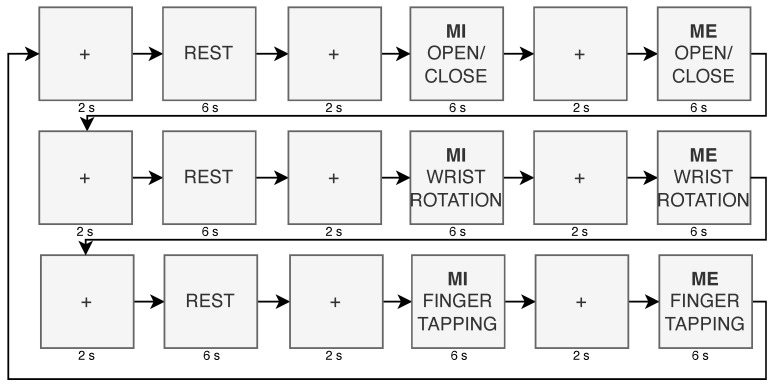
Instructions shown to participants for both MI and ME. The flow stops when 8 repetitions are reached.

Eleven healthy right-handed subjects (ten men and one woman; average age 28.27 ± 9.94) were recorded for 10 min. Before the recording, the consent information was signed by each of them. The protocol consists of a sequence of tasks, each composed of a series of actions shown on the screen to guide the participant in the movements (ME, MI, and rest). In Figure 5, the images depict the movements used in this work. Each action starts with a fixation cross displayed for two seconds, followed by an instruction to perform either imagined or executed movement shown for the subsequent six seconds. Each task is composed of a triplet of actions, with movements always presented in the same sequence: rest, MI, and ME. The protocol comprised a triplet of tasks, the first involving imagining and performing the open/close movement, followed by the imagined and executed wrist rotation, and finally the imagined and executed finger tapping. In Figure 6, structure of the protocol is reported and in Figure 7, the instructions are shown with one repetition of the protocol. Participants were shown the instructions on the screen and asked to continue performing the movement (MI or ME) until the next instruction appeared. Each triplet of all types of movement was repeated eight times, corresponding to 10 min. During the recording, the forearm was not sustained by any support, as shown in Figure 2.

### 2.3. Preprocessing Data

**Figure 8:**
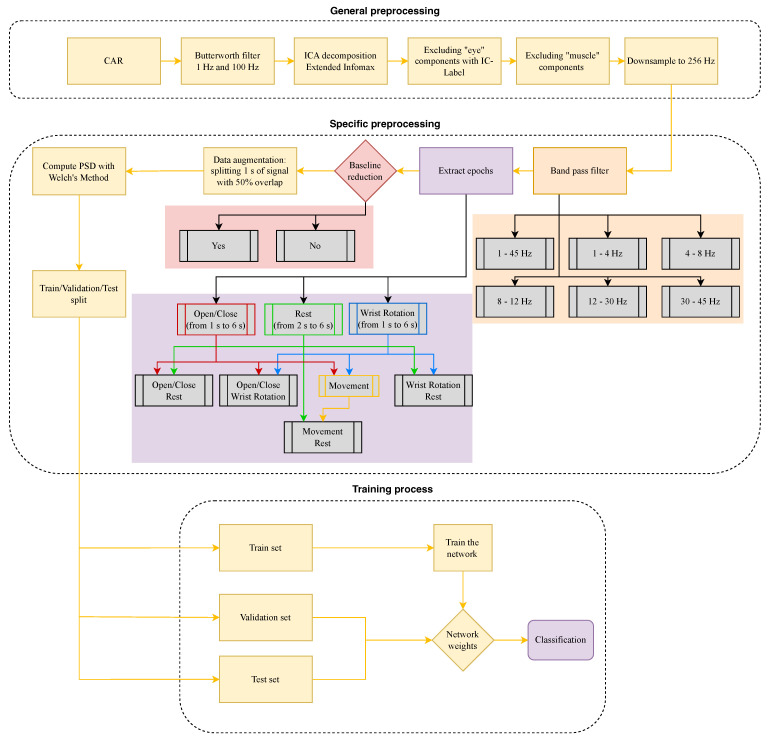
Preprocessing for sample generation and training. The colored boxes represent the parameters that change to explore different frequency bands (in orange) and the baseline reduction method (in pink), for each combination of movement/rest. The movement class (violet box) represents the merging of the open/close and wrist rotation. In the lower part of the scheme, the training process of the model is described.

The data were processed using the MNE [38] library for Python, and were divided into two parts: general preprocessing and specific preprocessing, as shown in Figure 8. The preprocessing pipeline started loading the channel location following the 10–20 international system. The first step of the general preprocessing included a Common Average Referencing (CAR) and a band-pass Butterworth filter between 1 Hz and 100 Hz. After that, the Independent Component Analysis (ICA) weights were computed using the Extended Infomax algorithm [39] with 500 iterations, and with IC-Label [40], the “eye” components with probability greater than 0.9 were removed. Subsequently, artifact removal was applied [41] (“muscle” components were removed with a probability greater than 0.9). The average of components for each subject identified as “eye” or “muscle” component removed are 0.2 and 1.3, respectively. Finally, the signal was downsampled at 256 Hz. For this study, neither MI classes nor ME of finger tapping movement were analyzed in depth.

**Figure 9:**
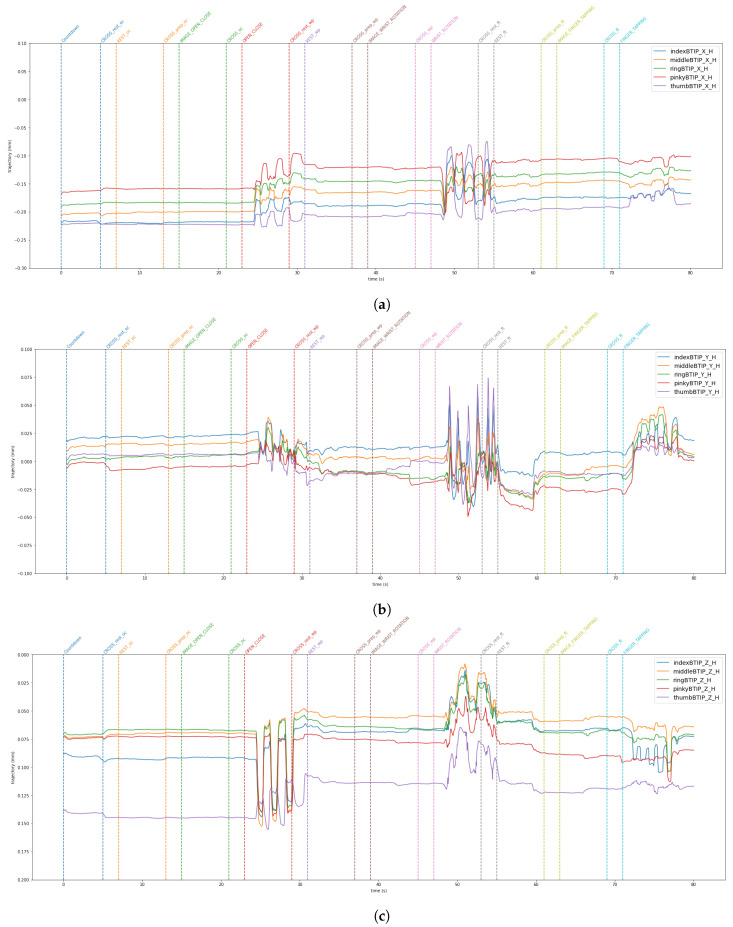
x (**a**), y (**b**), and z (**c**) components of the fingertip trajectory for the Horizontal LMC. All fingertips are reported in a single plot.

**Figure 10:**
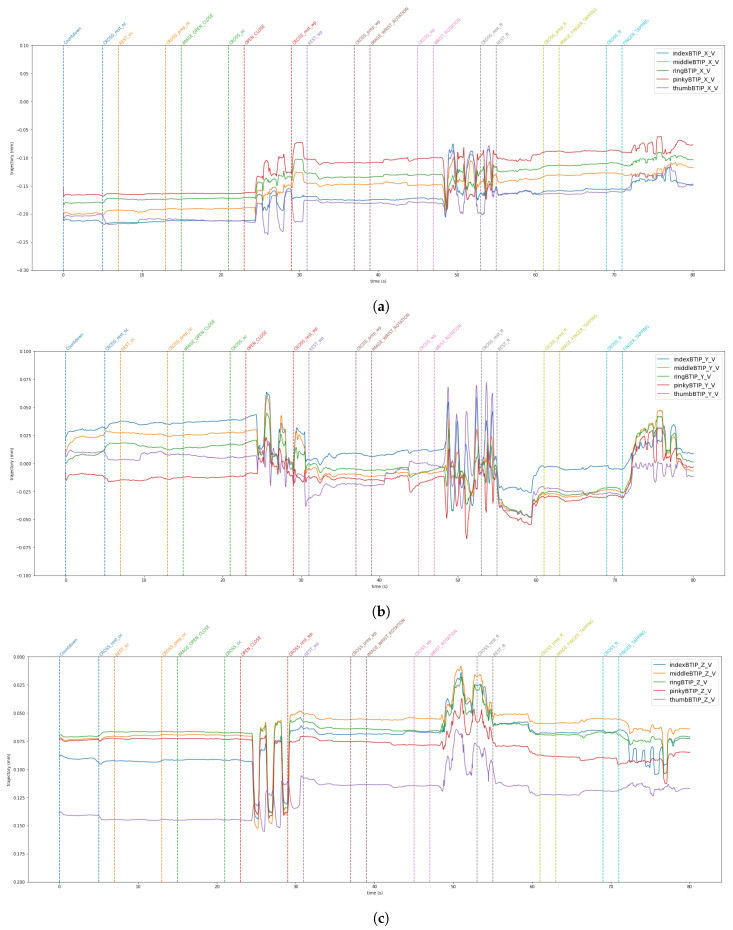
x (**a**), y (**b**), and z (**c**) components of the fingertip trajectory for the Vertical LMC. All fingertips are reported in a single plot.

**Figure 11:**
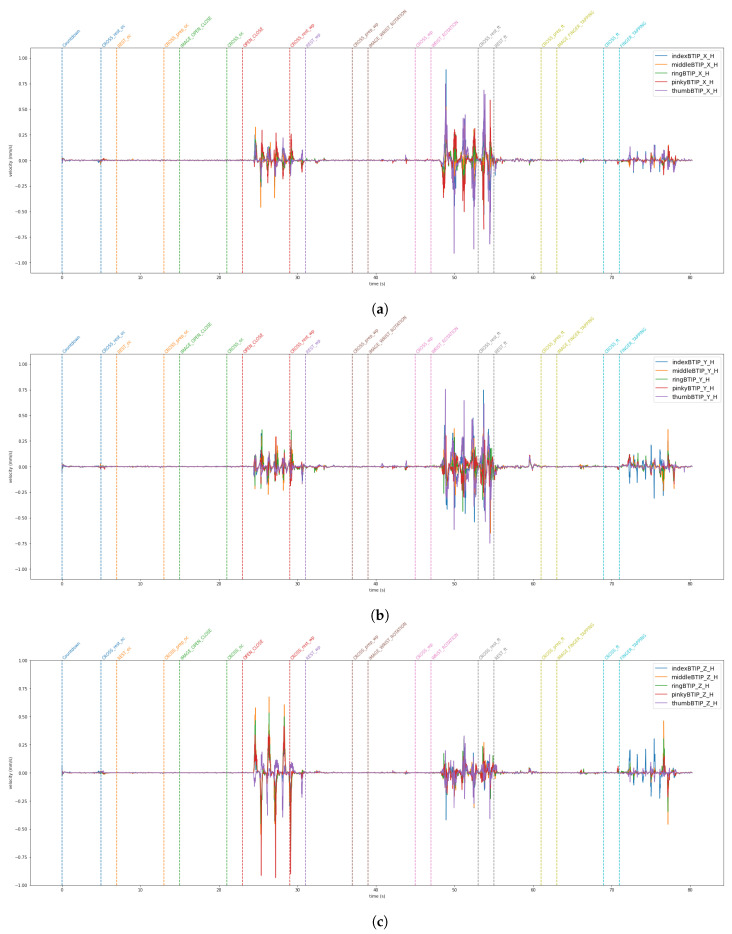
x (**a**), y (**b**), and z (**c**) components of the fingertip velocity for the Horizontal LMC. All fingertips are reported in a single plot.

**Figure 12:**
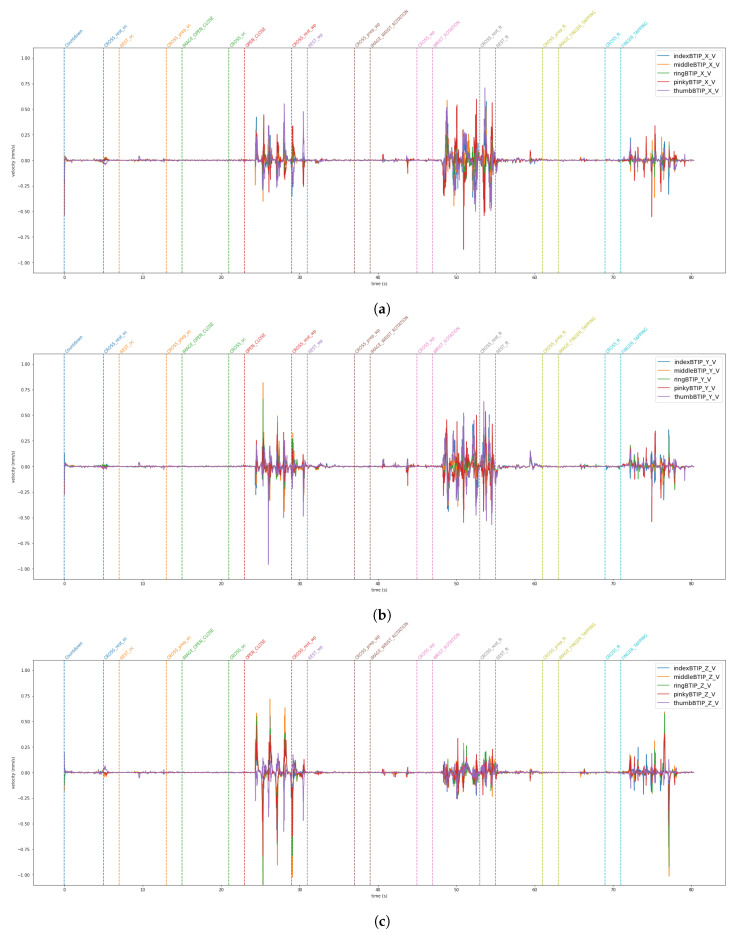
x (**a**), y (**b**), and z (**c**) components of the fingertip velocity for the Vertical LMC. All fingertips are reported in a single plot.

In the second part of the preprocessing, defining a specific preprocessing pipeline, a different band-pass filter was applied each time, and on the resulting signal, the baseline reduction method (subtraction of the EEG signal during fixation cross) was either applied or not. Furthermore, the epochs of the ME open/close, wrist rotation, and rest were extracted and combined in pairs. Moreover, in a further test, the two classes open/close and wrist rotation were combined to create a single class, “movement”, to see if it was more distinguishable from the rest condition. The result of these combinations (two baseline conditions, six bandpass filters, three movement classes, and one rest), corresponds to 46 trials. Then, the epochs aligned with the signal cue are segmented into pieces of one second, starting from the 2nd second for the open/close and wrist rotation epochs and the 3rd second for the rest epochs. The reason for this drop for the rest cue is to avoid interference with previous movements and to give time to start movements (open/close and wrist rotation). It was decided to exclude the 1st second for the movement because the movement did not start exactly with the appearance of the trigger but took a variable amount of time depending on the individual, as can be seen from Figure 9, Figure 10, Figure 11 and Figure 12. Furthermore, the participants reported they did not perform the first movement correctly. For the “rest” task, again based on the participants’ feedback and the analysis of the kinematic data, the first two seconds were excluded as the movement was not stopped instantaneously while the cross appeared but lasted for a few times even during “rest”. This new type of multi-modal dataset not only allows the extraction of possible patterns between the two modes, but also the preprocessing of data from one mode based on information extracted from another. In this specific case, the cut-off times of the EEG signal were chosen based on the kinematic data of the VG, without performing a combined analysis. Thus, the kinematic data made it possible to avoid biases due to the recording protocol, which could not have been avoided in the absence of kinematic data. During the epochs extraction after cutting the signals, a data augmentation process was applied extracting one second of signal shifting only by 0.50 s from the following epochs, overlapping each epoch with the following of 50%. Then, a train/validation/test split was performed to create the three sets for the neural network training, allocating 60%, 20%, and 20% of the original data, respectively. Finally, the PSD from each sample was computed using Welch’s Method [42] and used for training the network. Figure 8 shows the corresponding pipeline.

The data from the VG are stored in a JSON file. The LMC tracks hand movements in a 3D coordinate system. Using this raw data, the LMC software, Ultraleap Gemini v5.20.0, constructs a numerical model of the hand represented as a stick figure that mirrors the anatomy of the human hand, as illustrated in Figure 3. Specifically, the LMC data are structured into frames [37]. Each frame is a unique moment in time, and includes all of the captured data by the controller at that instance. Within each frame, details regarding detected hands are included. This encompasses information such as hand identifier, palm location (x, y, z coordinates), palm speed, palm orientation (the direction the palm is oriented towards), and palm trajectory (the path the palm is moving along). For the hand, comprehensive data regarding each finger is supplied. This consists of details like the finger’s unique identifier, type of finger (thumb, index finger, middle finger, ring finger, pinky finger), the position of the fingertip (x, y, z coordinates), finger’s direction, and velocity. Figure 9 and Figure 10 show the horizontal and vertical LMCs trajectory, respectively, and Figure 11 and Figure 12 show the horizontal and vertical LMCs velocity.

### 2.4. EEGnetV4

The EEGNetV4 [33] is a convolutional neural network (CNN) designed for raw EEG signals, in particular for BCI paradigms [43,44]. Its architecture is composed of depthwise and separable convolutions that allow the network to catch both spatial and temporal information [36]. Furthermore, this layer enables it to classify EEG signals by identifying common spatial patterns [45]. Moreover, EEGNetV4 can extract neurophysiologically interpretable features from the EEG signals it processes [33]. The main strength of EEGNetV4 is the ability to be trained with minimal data [46] demonstrating high decoding accuracy and shorter training times compared to other models [47]. In this work, the EEGnetV4 was chosen over other architectures, for its performance in classifying motion in public datasets [30].

## 3. Results

This section summarizes the analyses of the data acquired according to the procedures in the previous sections. Several frequency bands were analyzed, and the entire band [1–45 Hz] was filtered in the preprocessing phase to separate the sub-bands delta [1–4 Hz], theta [4–8 Hz], alpha [8–12 Hz], beta [12–30 Hz], and gamma [30–45 Hz]. Tests also include analyses with and without baseline removal. The classification step was accomplished using the EEGnetv4 implemented in the Braindecode Python library [25]. The block training process in Figure 8 shows the full training and testing process.

**Figure 13:**
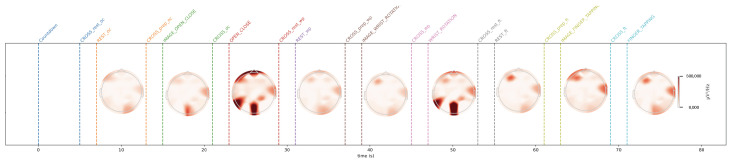
Spatial distribution of PSD for each task in a single subject. The timeline is the same as used in Figure 9, Figure 10, Figure 11 and Figure 12.

Table 1 illustrates the training hyperparameters, whose values were selected following extensive experimental trials. The Stochastic Gradient Descending (SGD) was used to increase training convergence, along with a dynamic Learning Rate (LR) that began at 0.004 and automatically dropped when there was no improvement after 25 idle epochs. The reduction factor for the LR was set at 0.2. Furthermore, we employed a double-value batch size [48], which was modified when the network showed no further enhancement even after the LR was reduced. Finally, we used a decay rate of 0.001 to boost the network learning rate. The change in the batch size throughout the learning phase enabled the network to improve its performance. For model evaluation, Accuracy, Precision, Recall, and F1 metrics were used. They are based on True Positive (TP), True Negative (TN), False Positive (FP), and False Negative (FN). Concerning rest vs. movement, the best result was obtained by analyzing the whole frequency range [1–45 Hz] with baseline reduction, achieving an accuracy of 0.64, whereas when analyzing rest vs. single movements, the frequency band that allows us to achieve better results was the theta band. The baseline reduction allowed us to achieve an accuracy of 0.61 and 0.55 for rest vs. open/close and open/close vs. wrist rotation, respectively. For rest vs. wrist rotation, the baseline reduction was not as effective as in the previous tests, obtaining an accuracy value of 0.58, above the chance value [49], but three percentage points less than the best result, shown in Table 2. To verify the robustness of the results obtained in the different combinations of tasks that exceeded the threshold [49], a five-group k-fold cross-validation was performed and the results are shown in Table 3. The stability in the average value and standard deviation confirms the robustness of the trained models. For the kinematic data acquired by the VG, the trajectories and velocities of each fingertip on each axis and for each of the two LMCs, horizontal and vertical, were calculated and reported in Figure 9, Figure 10, Figure 11 and Figure 12. From the graph of the z-component trajectories and velocities (the Cartesian reference system is oriented as in Figure 3), it can be seen that the more effective movements along this axis are open/close and finger tapping; while the wrist rotation movement is mostly affecting the x and y components. Observing the open/close z-component in the chosen time window, the individual performs on average 3 open/close movements. Regarding wrist rotation, the number of times a complete movement is performed is 3, as it is observed mostly on the y-axis. Regarding finger tapping, the most affected components are the y and z. All fingers are involved in finger tapping but the greatest contribution is by the index finger, mostly observable from the z-component. In the initial rest phase, the hand did not stop immediately, but took a variable time before arresting, depending on the individual and ∼1–2 s to maintain a constant position. Finally, for a preliminary visual analysis, Figure 13 reports the topoplots indicating the PSD distribution of one of the subjects in the dataset for each of the movements reported along the timeline. The analysis of the topoplots shows that the activation in certain areas is more pronounced in the ME task than in the MI task, and almost absent in the “rest” task.

## 4. Discussion

In this work, EEG signals and kinematic data related to MI and ME of open/close, wrist rotation and rest conditions were collected by 11 healthy subjects, stored in the MOVING dataset, and analyzed (those related to ME). Moreover, a new class called “movements” was built grouping open/close and wrist rotation, as shown in Figure 5. In the first step of the analysis, “rest” was chosen as the class of comparison between the two selected movements (merged in one class), in view of its usage with robot-assisted BCI. The tests carried out focused on finding the correct classification of the above-mentioned classes. Therefore, the following experiments were performed: rest vs. open/close, rest vs. wrist rotation, and open/close vs. wrist rotation. Despite the high-level noise affecting data (see Figure 1), using the preprocessing pipeline (Figure 8) and EEGnetV4 in the frequency domain, the results are above the literature threshold [49], as reported in Table 2, except in one case (open/close vs. wrist rotation). These results are in line with previous recent studies [30]. We also sought to determine which frequency band was more informative using PSD and found it was the theta band. Furthermore, the plots in Figure 9, Figure 10, Figure 11 and Figure 12 show the kinematics of the gestures used in this dataset for one of the analyzed subjects. By observing the trajectory and velocity, it is possible to reconstruct the movements of all the fingertips. The synchronism between the EEG data and the kinematic data also allows us to focus on part of the EEG signal with more information about the movement, as can be observed from the trajectories and velocities plots. It can also be noticed that the rest in the first few seconds is influenced by movements and, in the same way, the movement epochs in the first few seconds are in the rest position. In fact, before starting the execution, a subject-dependent delay, due to the processing of the stimulus, was present. The MOVING dataset, to the best of our knowledge, is the first dataset that contains a numerical model of the moving hand and the corresponding EEG motion-related signals. It aims to become the benchmark in the scientific community for multi-modal analysis of multi-modal signals produced by movement. Finally, from Figure 13, it can be observed that there is a gradual decrease in PSD in the transition from ME to MI and from MI to “rest”. This figure correlates with the cognitive effort required for the three tasks. One can see how the PSD between ME and MI presents the same spatial pattern, differing in the value of the PSD, as represented in the legend on the right. Moreover, the “finger tapping” ME does not show any movement-related pattern (Figure 13), and this result explains the difficulty of EEGnetV4 in classifying this task compared to “rest”, which on the contrary can be easily performed with the other two ME tasks. The examination of the PSD spatial distribution across various ME, MI, and “rest” conditions is conducted on a single subject to illustrate the potential analysis that can be carried out. This calculation excludes the first second of the ME/MI condition and the initial two seconds of the “rest” condition: this exclusion is determined based on kinematic data analysis and on feedback reported by subjects, which indicated when subjects began and finished movements. The MI and “finger tapping” tasks were not analyzed with EEGnetV4, but only used for representing their topoplots shown in Figure 13. Here, we provide additional data and tables that support the findings of this study.

## 5. Conclusions

This study aimed to introduce MOVING, a multi-modal dataset in a semi-controlled environment, consisting of EEG signals and kinematic data collected using VG during ME, MI, and rest conditions. The EEG data contained a high noise level due to dry electrodes: dry electrodes make the montage faster than wet electrodes. Furthermore, the influence of baseline reduction and frequency band analysis on ME classification was examined for only two ME movements and the rest condition, using PSD data extracted with Welch’s method. The results, reported in Table 2, show that, for rest vs. movement (open/close and wrist rotation) classification, the most informative frequency band is [1–45 Hz], and the classification is supported if the data are corrected by baseline reduction. For single movement detection, theta was the most informative frequency band for both movements, but the network performed better with the baseline reduction for rest vs. open/close, and without baseline reduction for rest vs. wrist rotation. Finally, the possibility of using the same network for the open/close vs. wrist rotation classification was also investigated, but the results were not above the chance [49]. Moreover, these findings are in line with previous studies that used the same conditions [30]. The kinematic data collected with the VG were also analyzed. The plots shown in Figure 9, Figure 10, Figure 11 and Figure 12 suggest that: (1) movements were not stopped immediately with the appearance of the trigger; (2) movements were not started as the start trigger appeared, but needed some latency; and (3) movements were well reconstructed based on trajectories and velocities. On these considerations, the EEG signals were cut before the analysis, to reduce the bias due to the late start or stop of the movements. These considerations could be useful for the design of future experimental protocols to contain cleaner, bias-free, multi-modal datasets with simultaneous recording of EEG signals and kinematic data. The topoplots representing the scalp PSD in the 1–45 Hz frequency band over the analyzed time interval (Figure 13) show that the activation of certain areas during ME (in two over three movements recorded) is more pronounced than during MI or “rest”. This initial analysis must be expanded by considering prior observations to conduct a comprehensive evaluation across all subjects. In conclusion, the MOVING dataset opens the possibility to investigate not only the preprocessing method and the characteristics of the hand movement extracted by the EEG signals, but also the relationship with the kinematic data acquired with the VG, here started with topoplot analysis. Future work will be dedicated to analyzing the MI part and building a portable system for safe operations in a work environment [50], improving the preprocessing steps by new fast algorithms [51,52], and investigating new models to analyze EEG data [53,54] to improve the performance classification in a real-life environment, such as to move a robotic arm in a dangerous workplace [55], or to interact with collaborative assistive devices [56,57]. Finally, in the future, the MOVING dataset will be increased by including more subjects (men and women, left- and right-handed subjects) to make it more robust.

## Figures and Tables

**Table 1:** The EEGnetV4 hyperparameters.

Hyperparameter	Value
Sample shape	1, 32, 129
Sampling rate	256 Hz
N. channels	32
N. epochs of training	2000
Optimizer	Stochastic Gradient Descending (SGD)
Scheduler type	Reduce learning rate when a metric has stopped improving
Learning rate	0.004
Patience	25 epochs
Factor scale	0.2
Batch size	16, 32
Decay	0.001
Baseline reduction	Yes/No

**Table 2:** Overview of the results. The movement class represents the merging of open/close and wrist rotation epochs. The best results for each combination of *baseline* and *filter* for each binary classification are in the box. In orange are the results that go above the chance threshold [49]. The box around the value indicates the best results for the classification.

*Baseline*	*Filter*	*Classes*	Precision	Recall	F1 Score	Support	Accuracy	*Classes*	Precision	Recall	F1 Score	Support	Accuracy
													
*Yes*	*1–45*	*rest*	0.71	0.51	0.59	346	0.64	*rest*	0.61	0.56	0.59	177	0.59
		*movement*	0.60	0.78	0.68	325		*open/close*	0.57	0.61	0.59	165	
*No*	*1–45*	*rest*	0.60	0.62	0.61	341	0.60	*rest*	0.54	0.73	0.62	165	0.57
		*movement*	0.60	0.58	0.59	334		*open/close*	0.64	0.43	0.52	182	
*Yes*	*1–4*	*rest*	0.54	0.7	0.61	346	0.54	*rest*	0.52	0.68	0.59	177	0.51
		*movement*	0.53	0.37	0.44	325		*open/close*	0.49	0.33	0.39	165	
*No*	*1–4*	*rest*	0.54	0.64	0.59	341	0.55	*rest*	0.50	0.71	0.58	165	0.52
		*movement*	0.55	0.45	0.50	334		*open/close*	0.57	0.35	0.44	182	
*Yes*	*4–8*	*rest*	0.60	0.57	0.58	389	0.57	*rest*	0.64	0.55	0.59	190	0.61
		*movement*	0.55	0.58	0.57	358		*open/close*	0.58	0.67	0.62	179	
*No*	*4–8*	*rest*	0.58	0.56	0.57	382	0.58	*rest*	0.51	0.63	0.57	183	0.53
		*movement*	0.57	0.59	0.58	379		*open/close*	0.56	0.44	0.49	195	
*Yes*	*8–12*	*rest*	0.56	0.51	0.53	403	0.54	*rest*	0.53	0.68	0.60	191	0.55
		*movement*	0.52	0.56	0.54	376		*open/close*	0.59	0.43	0.50	201	
*No*	*8–12*	*rest*	0.57	0.48	0.52	414	0.54	*rest*	0.53	0.51	0.52	191	0.54
		*movement*	0.51	0.60	0.55	369		*open/close*	0.55	0.57	0.56	201	
*Yes*	*12–30*	*rest*	0.58	0.60	0.59	405	0.57	*rest*	0.54	0.71	0.61	198	0.55
		*movement*	0.56	0.55	0.56	383		*open/close*	0.57	0.39	0.46	198	
*No*	*12–30*	*rest*	0.58	0.52	0.55	405	0.56	*rest*	0.52	0.71	0.60	198	0.53
		*movement*	0.55	0.61	0.57	383		*open/close*	0.55	0.35	0.43	198	
*Yes*	*30–45*	*rest*	0.50	0.32	0.39	405	0.48	*rest*	0.48	0.55	0.51	198	0.47
		*movement*	0.48	0.66	0.55	383		*open/close*	0.47	0.39	0.43	198	
*No*	*30–45*	*rest*	0.52	0.44	0.47	405	0.50	*rest*	0.48	0.62	0.54	198	0.48
		*movement*	0.49	0.57	0.52	383		*open/close*	0.47	0.34	0.39	198	
*Yes*	*1–45*	*rest*	0.65	0.49	0.56	172	0.60	*open/close*	0.52	0.58	0.55	128	0.53
		*wrist rotation*	0.56	0.71	0.63	157		*wrist rotation*	0.53	0.48	0.50	128	
*No*	*1–45*	*rest*	0.57	0.53	0.55	172	0.54	*open/close*	0.50	0.43	0.46	128	0.50
		*wrist rotation*	0.52	0.56	0.54	157		*wrist rotation*	0.50	0.58	0.54	128	
*Yes*	*1–4*	*rest*	0.58	0.63	0.61	172	0.57	*open/close*	0.56	0.45	0.50	128	0.55
		*wrist rotation*	0.55	0.50	0.52	157		*wrist rotation*	0.54	0.65	0.59	128	
*No*	*1–4*	*rest*	0.58	0.60	0.59	172	0.56	*open/close*	0.49	0.46	0.48	128	0.49
		*wrist rotation*	0.54	0.52	0.53	157		*wrist rotation*	0.49	0.52	0.51	128	
*Yes*	*4–8*	*rest*	0.56	0.64	0.60	183	0.58	*open/close*	0.56	0.67	0.61	153	0.55
		*wrist rotation*	0.61	0.53	0.57	195		*wrist rotation*	0.54	0.43	0.48	141	
*No*	*4–8*	*rest*	0.57	0.72	0.63	180	0.61	*open/close*	0.55	0.38	0.45	159	0.51
		*wrist rotation*	0.67	0.51	0.58	203		*wrist rotation*	0.48	0.65	0.55	139	
*Yes*	*8–12*	*rest*	0.52	0.51	0.52	194	0.52	*open/close*	0.53	0.61	0.56	157	0.51
		*wrist rotation*	0.52	0.53	0.52	193		*wrist rotation*	0.49	0.41	0.45	144	
*No*	*8–12*	*rest*	0.52	0.50	0.51	191	0.53	*open/close*	0.52	0.24	0.33	164	0.47
		*wrist rotation*	0.54	0.56	0.55	201		*wrist rotation*	0.46	0.74	0.56	141	
*Yes*	*12–30*	*rest*	0.54	0.55	0.55	191	0.55	*open/close*	0.57	0.49	0.53	164	0.52
		*wrist rotation*	0.57	0.56	0.56	201		*wrist rotation*	0.49	0.56	0.52	141	
*No*	*12–30*	*rest*	0.53	0.48	0.50	191	0.54	*open/close*	0.55	0.40	0.46	164	0.50
		*wrist rotation*	0.55	0.59	0.57	201		*wrist rotation*	0.47	0.62	0.53	141	
*Yes*	*30–45*	*rest*	0.45	0.42	0.43	191	0.47	*open/close*	0.57	0.45	0.50	164	0.52
		*wrist rotation*	0.48	0.51	0.50	201		*wrist rotation*	0.49	0.60	0.54	141	
*No*	*30–45*	*rest*	0.46	0.52	0.49	191	0.47	*open/close*	0.55	0.42	0.48	164	0.50
		*wrist rotation*	0.48	0.42	0.45	201		*wrist rotation*	0.47	0.60	0.53	141	

**Table 3:** Results performing 5-fold cross-validation on the best models reported in Table 2.

k-Fold	Rest/Mov	Rest/Wrist	Rest/Openclose
1-fold	0.59	0.63	0.61
2-fold	0.62	0.61	0.60
3-fold	0.62	0.58	0.61
4-fold	0.60	0.59	0.60
5-fold	0.64	0.61	0.61
*average* ± dev.st	*0.61 ± 0.02*	*0.60 ± 0.02*	*0.61 ± 0.01*

## Data Availability

The dataset is available at: https://zenodo.org/records/12804784, accessed on 20 June 2024.
